# Family carer experiences of hospice care at home: Qualitative findings from a mixed methods realist evaluation

**DOI:** 10.1177/02692163231206027

**Published:** 2023-10-21

**Authors:** Vanessa Abrahamson, Patricia Wilson, Stephen Barclay, Charlotte Brigden, Heather Gage, Kay Greene, Ferhana Hashem, Rasa Mikelyte, Melanie Rees-Roberts, Graham Silsbury, Mary Goodwin, Brooke Swash, Bee Wee, Peter Williams, Claire Butler

**Affiliations:** 1 Centre for Health Services Studies, University of Kent, Canterbury, UK; 2 University of Cambridge, Cambridge, UK; 3 Pilgrims Hospices, Canterbury, UK; 4 Department of Clinical and Experimental Medicine, University of Surrey, Guildford, UK; 5 Mary Ann Evans Hospice, Nuneaton, UK; 6 Lay Author (Member of the Public); 7 University of Chester, Chester, UK; 8 Oxford University Hospitals NHS Foundation Trust and Harris Manchester College, Oxford University, Oxford, UK; 9 Department of Mathematics, University of Surrey, Guildford, UK

**Keywords:** Hospice care, hospice and palliative care nursing, palliative care, terminal care, bereavement, caregivers, caregiver burden, health services research.

## Abstract

**Background:**

Hospice-at-home aims to enable patients approaching end-of-life to die at home and support their carers. A wide range of different service models exists but synthesised evidence on how best to support family carers to provide sustainable end-of-life care at home is limited.

**Aim:**

To explore what works best to promote family carers’ experiences of hospice-at-home.

**Design:**

Realist evaluation with mixed methods. This paper focuses on qualitative interviews with carers (to gain their perspective and as proxy for patients) and service providers from 12 case study sites in England. Interviews were coded and programme theories were refined by the research team including two public members.

**Setting/participants:**

Interviews with carers (involved daily) of patients admitted to hospice-at-home services (*n* = 58) and hospice-at-home staff (*n* = 78).

**Results:**

Post bereavement, 76.4% of carers thought that they had received as much help and support as they needed and most carers (75.8%) rated the help and support as excellent or outstanding. Of six final programme theories capturing key factors relevant to providing optimum services, those directly relevant to carer experiences were: integration and co-ordination of services; knowledge, skills and ethos of hospice staff; volunteer roles; support directed at the patient–carer dyad.

**Conclusions:**

Carers in hospice-at-home services identified care to be of a higher quality than generic community services. Hospice staff were perceived as having ‘time to care’, communicated well and were comfortable with dying and death. Hands-on care was particularly valued in the period close to death.

**What is already known about this topic?**

- Increasingly, people at end-of-life want to die at home but this relies heavily on family carers to support the patient.
- Many carers struggle with the practical and emotional burden of caring for a loved one at home.
- Services providing hospice care at home are highly rated by carers but access is limited and the model of care varies greatly between services, with little data on how this affects patient/carer experiences.

**What this article adds?**

- Hospice-at-home services need to set clear expectations from the start so that families know exactly what the service can, or cannot, provide; this helps establish confidence in the service and build a strong relationship with the carer.
- Carers valued the expertise of hospice staff (in death and dying) and that they had time to care in a flexible and compassionate manner, which other services lacked.
- Carers felt ‘doubly’ bereaved when the person they cared for died and the hospice team immediately withdrew; existing bereavement services did not suit many carers, particularly younger families.

**Implications for practice, theory or policy**

- Carers appreciated early contact with services but placing the onus on carers to trigger increased help when needed was not found supportive.
- There should be regular review of needs for the carer as well as for the patient and services available to address both their needs; services could consider options to increase volunteer contributions to hospice-at-home services.
- Hospice services could consider how to provide bereavement support that meets carer preferences.

## Introduction

National strategy in England^1,2^ supports the drive towards encouraging choice and increasing opportunities to be cared for and die at home and appears aligned with public preferences.^3^ The World Health Organisation^4^ recognised that people in need of palliative care prefer to remain at home, and to address this preference, palliative care programmes should be incorporated into existing healthcare systems to enable end-of-life care to be accessible in patients’ homes. Estimates of how many people die at home vary widely. For example, in 2019, only 24% of all deaths in England occurred at home (not including care home deaths)^5^ with most older people unable to access hospice care, instead reliant on statutory services which lack the capacity and expertise to meet this demand.^6^

Hospice-at-home services are a sub-set of designated palliative care services, often linked to a local hospice organisation (and building) and aim to offer the quality and ethos of hospice care at home to support dying patients to have a ‘good death’ and to meet their preferences. Most are small organisations and must raise about two-thirds of their own funding.^7^ Additionally, home-based care is heavily dependent on family/informal carers having resources and skills to provide unpaid care.^8^ Our realist review identified wide variation in hospice-at-home models of care in the United Kingdom^9^ and studies reported such a range of outcome measures that it was not possible to make useful comparisons. It was also unclear what elements of hospice-at-home services delivered what outcomes and to what extent such outcomes were delivered in conjunction with other services. This lack of clarity makes sharing good practice difficult and limits evidence-based service improvements. Subsequently, our realist evaluation asked ‘What are the features of Hospice-at-home models that work, for whom and under what circumstances?’^10^ Given the breadth of the evaluation, this paper focuses on carer perspectives because their insights, as the patient’s proxy at a time when patients were often too unwell to interview provided key data, are integral to improving frontline care and service development. This paper asks:

1. What were family carers’ experiences of hospice-at-home care?
2. What did good support look like and how was it achieved?

## Methods

### Overall study design

*Op*timum Models Of Hospice-at-home Services For *E*nd-Of-*L*ife Care (OPEL) employed a mixed methods design using realist evaluation methodology.^10^ Realist methodology aims to evaluate complex interventions in the real-world setting. Evidence is presented as programme theories (key features of the service with a description of what mechanisms appear to be leading to certain outcomes).^11^ These programme theories are supported by details of the context (C), mechanisms (resources and responses of those delivering and accessing the service) (M) and the intended (and unintended) outcomes (O), referred to as CMO configurations.^11^

**Figure 1.**
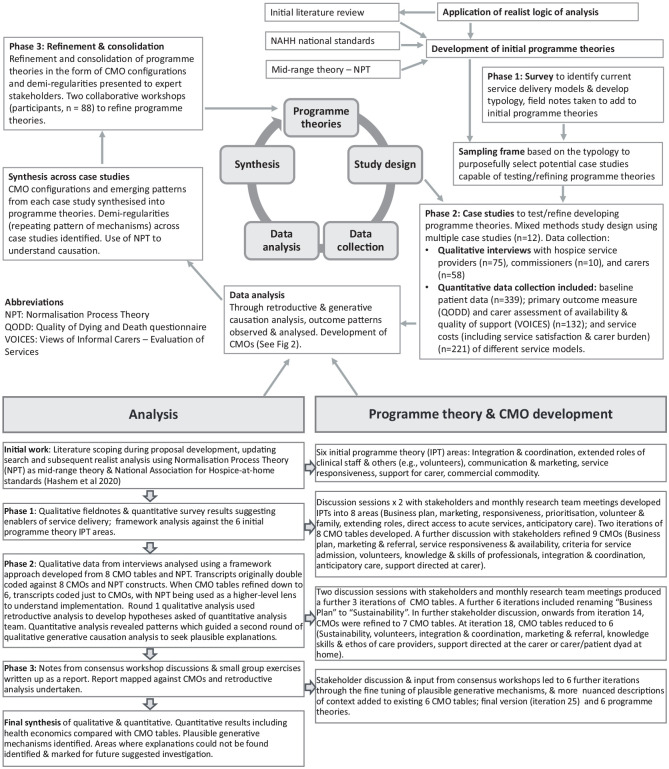
Study design, data analysis and theory development.

The study comprised three phases (Figure 1).^12^ The National Association for Hospice-at-home standards^13^ were used, in conjunction with our literature review^9^ and Normalisation Process Theory^14^ (NPT) as mid-range theory and conceptual framework to build initial programme theories (how hospice-at-home should work). The initial programme theory pertaining to carers is presented in Table 1. All initial programme theories informed Phase 1, a national survey of hospices which aimed to describe key features of hospice-at-home services.^15^

**Table 1.** Initial programme theory on Support Directed at the Carer.

Support Directed at the Carer
For carers to be able to continue to provide sustainable care at home to partners or family, and for services to understand what carers themselves may need to sustain them, a holistic understanding of carers’ needs including an assessment by a multidisciplinary team involving the carer and hospice-at-home staff is needed for carers to receive the practical and emotional support, to be mentored to use equipment, undertake key tasks and receive support through a crisis.

Phase 2, case studies, is described below with further information available.^10^ Phase 3 comprised two national consensus workshops to validate and consolidate the programme theories.^10^

### Case study design and site selection

Phase 1, a national telephone survey of seventy Hospice-at-home services in England, was carried out to develop a typology of service characteristics and a sampling framework for recruiting case study services for Phase 2.^15^ Case study design is an established approach to conducting research in ‘real-life’ healthcare settings and compatible with a realist orientation.^16^ Each case, or unit of analysis, was defined as what the site described as their hospice-at-home service. Services were categorised into four models based on size (large vs small, with a cut-off of 365 referrals/year) and whether (or not) a service provided care 24/7. We purposively sought diversity in geographical spread, socio-economic profile, staffing mix and funding sources. Twelve case study sites were recruited from those who expressed interest in the Phase 1 survey. The 12 sites were allocated across the four models and the number of sites in each model was based on their activity levels, to ensure that recruitment could meet the required numbers to fulfill quantitative/health economic data collection.

#### Sampling and recruitment

Convenience sampling within sites and during a set time period was used to identify patient-carer dyads, who were invited by hospice staff to participate in the research when they were admitted to the service. Inclusion criteria were patients who had an informal/family carer (defined as someone who provided daily care and support at home) who also agreed to participate and was able to give informed consent.

Relevant training about the study, recruitment and the informed consent process was provided to site staff (research nurses, clinical staff, managers) by members of the research team. The training was delivered in person at site initiation visits with follow-up training as needed.

Hospice managers identified relevant potential staff interviewees who were then recruited directly by the study team.

### Data collection

Staff interviews (face-to-face or telephone) used a topic guide informed by realist thinking (Supplemental Material 1), including questions about service history, funding, processes/contextual features affecting service delivery and enablers/barriers to providing hospice care at home.

A mixed methods approach collected quantitative data comprising information about the patient and informal/family carer on admission to hospice-at-home and outcome measures from carers post-bereavement; qualitative interview data (described here) and carer collected health economics data.^10^

Post-bereavement, carers were asked two short questions about the overall care received, at 6-months post-bereavement (as determined by our lay advisors and ethics committee). The first asked if the carer and family had received as much support as they needed when caring for the patient (five-point scale, from ‘as much as needed’ to ‘no help at all’); the second was a rating of the quality of care received (five-point scale, from ‘outstanding’ to ‘poor’) (Supplemental Material 2). These questions were taken from the Views of Informal Carers – Evaluation of Services (VOICES) survey Short Form (an annual national survey designed to look at the quality of end-of-life).^17^ After questionnaire completion, all carers were invited to an optional interview.^19^ Telephone interviews followed a topic guide informed by realist thinking and NPT (Supplemental Material 3), exploring carers’ experience of the service and end-of-life care. Given the sensitivity of the topic, we opted for a conversational style, as others have done in realist studies,^18^ allowing carers time to think around the question and focus on what they considered most important. The researchers had a comprehensive understanding of developing CMO configurations and were able to probe with sensitivity for ‘nuggets’ of information^19^ appertaining to our developing programme theories. We aimed to interview up to 20 carers per service model type, a pragmatic decision. Over the many months of data analysis, we reached a stage where we could not identify any new data to further our CMOs. We analysed data within each hospice site, within each model, and across the whole data set.

### Qualitative data analysis

Data analysis (Figure 1) was undertaken using retroductive data analysis.^20^ Retroduction demands counterfactual thinking based on knowledge and experience, analysing why expected phenomena anticipated in initial programme theories may or may not be present, and identifying what conditions are needed for them to be triggered.^20^

Prior to monthly team meetings, all team members, including two public members, read and annotated a batch of transcripts. We then discussed these in relation to NPT constructs and our developing CMOs, thus enhancing rigour. Research assistants also coded transcripts in NVivo which provided an audit trail. We focused on programme theory areas, trying to tease out CMOs in each area. CMOs are embedded components of a programme theory that explain why aspects of interventions work, in particular circumstances and to what extent.^21^ We had site-specific transcript meetings (e.g. all Hospice A), model specific meetings (e.g. all small sites providing 24/7 care) and mixed sites/models. We kept a running document (‘CMO Configuration table’) on which all ideas about developing CMOs were documented, with the code of relevant transcript and direct quotes/page numbers. Table 2 provides an example.

**Table 2.** Example of transcript coding.

*Interviewer: And was that night-time service you used?* Yeah. We had to phone at about 2 o’clock in the morning [*CMO3 Service Responsiveness & Delivery, C = 24/7 service*] they were magnificent . . . it was very clear that every time we phoned the person that we spoke to was really knowledgeable about [husband]’s case. [*CMO6 Knowledge & Skills of Care Providers, M = knowledge & expertise*] *Interviewer: Right. So that made you, helped you feel reassured?* Yeah actually because. . . I don’t think I realised at the time how stressful that was but it is, and of course, as the carer you’re the one having to deal with it. So it was really reassuring for both the patient and the carer [*CMO9 Support Directed at the Carer, M = reassurance*], they were saying ‘no, this sounds as though you might need this, I think, don’t worry about that, give it time to settle, just top up the Morphine but just keep a note of how much because that’s important for your longer term care [*CMO6 Knowledge & Skills; CMO8 Anticipatory Care, M=overview/expertise*] and, don’t worry, this does often happen’ or ‘no, you really need, I think we need to get you a GP visit’ [*CMO7 Integration and Co-ordination, M=good working relation with GP*] and whatever. (PC34, wife)

Additionally, findings were regularly discussed with lay and content expert stakeholders, and developing hypotheses tested in subsequent batches of transcripts. Common patterns (‘demi-regularities’)^22^ within the data were used to refine/justify emerging theories.

The CMO Configuration table went through 25 iterations over 18 months (Supplemental Information 4) and described common patterns that could be applied to different settings and in particular, the generative mechanisms at work. The final version was consolidated at the Phase 3 consensus event.^10^

### Ethical considerations

Health Research Authority governance and ethics approval was granted in 2017 (London Queen Square Research Ethics Committee, REC reference 17/LO/0880, IRAS project ID: 205986). We were cognisant of the vulnerability of the subjects and the study had a distress protocol. A variable, written consent process was used, including a personal/nominated consultee process, for patients lacking capacity. No data were collected directly from patients. Gate-keeping by staff due to patient vulnerability was a concern for which we offered training and support throughout.

### Patient and Public Involvement (PPI) and stakeholder engagement

Stakeholder involvement is a key feature of realist methodology. By engaging lay and content experts, evidence is built to support theories based on coherence and plausibility.^23^ Stakeholder involvement was operationalised through public team members who were closely involved throughout from study inception to completion, 6-monthly meetings with the project oversight group which included lay and content experts, consultation with patients from a hospice which was not a case study site and two national consensus workshops.

## Results

### Sample description

All interviews were carried out over 18 months between 2018 and 2020. We recruited 339 patient/carer dyads of whom 284 patients (83.8%) died within the study period. Fifty-eight carers were interviewed of whom the majority were female (70.2%) and the spouse/partner of the patient (60.4%); most patients had a diagnosis of cancer (76.8%), a long-standing and persistent feature of UK hospice and palliative care services.^24^ There were no differences in background socio-demographic characteristics of participants recruited across the four models.^10^

Service providers included frontline staff (healthcare assistants, nurses), middle-management (e.g. service leads, volunteer coordinators, fundraising managers), senior managers/executives. Table 3 provides interview numbers per model and per case study site.

**Table 3.** Number of interviews by interviewee group and case study site.

	Case study site (pseudonym)	Carer	Service provider^a^	Total per model
Large 24/7	A	0	5	
	C	1	4	
	E	7	6	
	P	20	5	
		28	20	48
Small 24/7	D	7	6	
	G	3	9	
	L	3	8	
	V	4	6	
		17	29	46
Large not 24/7	W	4	8	
		4	8	12
Small not 24/7	H	2	4	
	M	5	7	
	X	2	8	
		9	19	28
Total		58	76	134

a Seventy-six interviews with 75 service providers (3 of whom were interviewed twice and 4 of whom took part in interviews as a pair).

**Figure 2.**
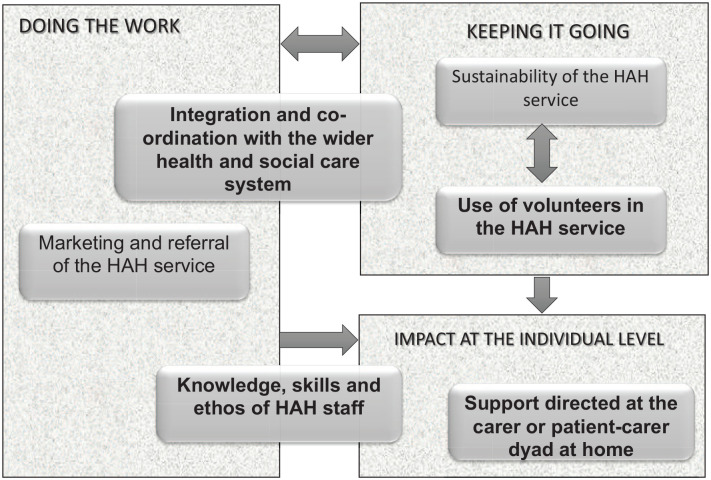
Relationship between final programme theories.

The final iteration of six programme theories (Table 4) captured factors relevant to providing optimum hospice-at-home services. Figure 2 demonstrates how programme theories were interrelated; data for this paper is drawn from four areas (shown in bold) which related most closely to the patient/carer experience. We have not drawn from two programme theories (marketing and sustainability) because carers rarely commented on these aspects. Full details of all CMO configurations and final programme theories are available as (Supplemental Information 4 and 5 respectively). Illustrative quotes indicate the site (by letter); role (C for carer and SP for service provider) and each interviewee’s unique identifier.

**Table 4.** Final programme theory on Support Directed at the Carer.

Support Directed at the Carer or patient-carer dyad at home
Unpaid care provided by family and friends is critical to enable patients to remain at home. How the patient and their informal carer, as a unit in the home, feel about dying at home and respond to the challenge of this situation will be key to achieving death at home. The carer may require confidence and new skills to enable them to provide care up to and including the point of death at home. In bereavement, there may be short or long-term consequences of caring to the carer’s mental and physical health. However, there is a concern about medicalising bereavement which is a normal process. A full assessment of care needs including the whole family/care unit is required. The hospice-at-home service fully informs the carer about what might happen in terms of the trajectory of illness and the increasing burden of caring over time. Carers will then know what to expect and can rapidly recognise a change in caring situation from control to crisis. If carer and patient choices are affirmed and supported wherever possible, the carer and patient have an increased sense of control. The hospice-at-home service should negotiate a partnership with the carer, including clarity about what can and cannot be provided, and recognition of what the patient-carer dyad wants. Pre- and post-bereavement support is based on relationship and understanding of the situation, and a shared story of caring for the patient. In addition, those not experiencing normal bereavement need to be recognised and additional help made available. This should not rely on self-referral and the timing may be many months post bereavement.

To contextualise the qualitative findings, responses to the VOICES-Short Form found that most carers across all sites (97/127; 76.4%) thought that they had got as much help and support as they needed in the period before the patient died. Similarly, most carers (97/128; 75.8%) rated the help and support they received as excellent or outstanding.^10^ Quantitative and health economics data outlined differences between models and sites but qualitative data was surprisingly consistent in relation to this paper’s aims so we have focused on key findings, pertinent to all services, only commenting on model/site variations as appropriate.

### Integration and co-ordination

Integration and co-ordination were integral to ‘doing the work’ of hospice-at-home services across all case study sites. National policy had impact, largely through influencing commissioning decisions, but most relevant to patient/carer experiences were the local strategies hospices had taken to enhance integration and co-ordination. At an organisational level, a blended approach enabled different services to provide what was needed without hard boundaries between services, for example using honorary contracts with the NHS, shared clinical records, single point of access arrangements (and co-location) and systems to decrease bureaucracy. At an operational level, initiatives to build relationships included joint home visits, secondments across settings to learn about each other’s role and regular inter-organisational meetings:

We have a quarterly End of Life Community Nurse Meeting which we all go to, so the CNSs, the Hospice-at-home Team, the Community Nursing Services all go too and that helps to iron out what some of the crossover and communication issues are. . . . . . So we maintain a daily, open dialogue and then we have the quarterly meetings where we all get together and talk about if we’ve got any issues. MSP05 (Integrated Community Team Leader for End-of-life Care)

Some services agreed on a division of labour, based on ‘finding the best person to do the job at the time’ (XSP04, community matron). This usually stemmed from bottom up, when hospice-at-home staff worked closely with colleagues in partner organisations to enhance joint working.

Family carers were often responsible for co-ordinating care prior to hospice involvement and were relieved when hospice staff took over responsibility, as with this nurse talking about her sister:

Imagine what was going through my mind on that Friday morning when, I thought, well we can’t leave her like this over the weekend. . . They’d [hospital staff] given her no pain relief. . . other family members were looking to me in absolute confusion. . . She was just discharged from hospital with nothing. . . . . . . Hospice-at-home came out on the Saturday. . . . . . once we’d got Hospice-at-home in, everything was taken care of. DC31 (sister)

Carers particularly valued out-of-hours support (24/7 sites):

The nurse at the end of that phone said to me ‘now you do realise we have a 24 hour helpline at the hospice, if you have any queries, any problems whatsoever just pick up the phone and someone is here’. . . I put the phone down and I burst into tears because it was the first time I felt we were being truly supported to care for mum. DC21 (daughter)

Most carers knew who, or which service, to contact for what but there were examples where carers were uncertain, leading to delayed access to appropriate care. For example, this couple received monthly monitoring calls from their hospice:

During the day, she was in pain. . . I wasn’t quite sure who we should call so we phoned the hospice and they said ‘well, I think you better call the doctor or ring was it, 111 or something like that? . . . eventually the ambulance took her to the hospital. . . she never came out. . . she did want to go to the hospice but things didn’t turn out that way’. EC05 (husband)

### Knowledge, skills and ethos

Hospices valued their status as experts and family carers consistently commented on the quality of care. Hospice-at-home staff were highly skilled (whatever discipline or level of training) and were differentiated from others by their ethos:

Everyone we met from the hospice throughout those three and a half months. . . all had that wonderful, wonderful ethos. DC21 (daughter-in-law)

Alongside their expertise and professionalism:

[Hospice] carers were absolutely gold standard excellent, and from agencies it was hugely variable. PC12 (wife)

We had to phone at about 2 o’clock in the morning. . . they were magnificent. . . . . . it was very clear that every time we phoned the person that we spoke to was knowledgeable about [husband]’s case. . . . . . it was really reassuring for both the patient and the carer. PC34 (wife)

A frequent metaphor was that hospice-at-home staff had time to care, not just temporal but also the perception that they were flexible and able to offer individualised patient led care, at the pace of the patient-carer dyad:

I just feel time is such a massive, massive factor. And that just allows people to open up more when we’re there each day. They can see that there’s no rush. They can see that we want to give quality care. . . time is so massive. It’s priceless isn’t it, time. MSP01 (hospice-at-home sister)

This included being experienced in and comfortable with death/dying which manifested in supporting the patient-carer dyad and providing hands-on care (often small healthcare-assistant led services), particularly important close to death:

The [hospice-at-home] nurse was there. . . she was. . . sort of preparing us and saying. . . his breathing’s changed again and you know, we went through the process. And it was sort of nice and reassuring that, you know, she was almost like forewarning us so that we could be there with me dad right to the very end. DC11 (daughter)

However, there were examples when care was provided over an extended period (depending on the hospice service but usually larger services) and consistency of care was affected:

In the last months she [wife] then had to readjust that you had constantly changing hospice personnel arrive. . . you had exactly the same with the [statutory] carers. . . there was no continuity. EC14 (husband)

### Support directed at the carer

Successful care at home depended heavily on the family carer. In strong services care was moulded around the dyad, combining co-ordinated processes with high quality care. Notable was the use of ‘family’ as metaphor, used by both carers and staff:

I think distinctive was it just made me feel like it was personal to us and I felt comfortable, almost like a family. . . you feel cocooned in this world of they’re there to help you and I felt comfortable and just the whole thing helped us a lot. GC18 (wife)

Moulding care around the dyad also involved equipping carers with the necessary knowledge and skills to develop confidence in their own abilities. One hospice ran a carers’ course which included sessions on mobility, nutrition, finances and the process of dying which most found helpful:

The mobility one was very good, which was teaching us how to use the slidey sheet and how to get people out of a chair and the Sara bedding equipment. EC06 (husband)

However, it was crucial that staff set realistic expectations with the family at the beginning and continued to communicate openly/honestly about what the service could, or could not, provide. Most carers were relieved to have whatever support was offered but some felt there was an inherent pressure to support dying at home as the preferred option:

It’s very seductive to say to somebody do you want to die at home. Even quite late. . . a week or so into her time in the hospice and they asked her again ‘do you want to die at home?’ and she said ‘yes’ and that was the moment when we [daughters] thought ‘oh my god, now we’re going to have to prepare full care. . . we will not be able to cope with this stage’. XC01 (husband)

Carers had not anticipated how this would change their role and affect the family:

You don’t understand certainly at the beginning what the scale of the task is going to be as a carer. . . it caused. . . tremendous upset really. She [wife/mother] came to resent us as her carers and yet we’re trying to be the loved ones. . . the role between the carer and the relative gets quite confused. XC01

Another expectation was around the available length of involvement which varied considerably between sites from 2 weeks to over 1 year, depending on their service model and referral criteria. Carers often expected the service to last as long as needed, but for patients who took ‘too long to die’, withdrawing care was shocking:

I didn’t realise that it [hospice-at-home] only lasted for so long, something like two weeks. . . all of a sudden there was a knock at the door and it was some other carers and I didn’t know anything about it, and neither did the two [hospice-at-home] girls who happened to be in on the morning seeing to him. . . Evidently, they only do it for so long and then it changes over. I didn’t know that and it upset me. CC13 (wife)

Similarly, carers expected a hospice bed to be available when needed but this was not always possible:

The ultimate sadness with [hospice] was that [husband] died in hospital, he didn’t die in hospice and. . . I called every day of that last week saying ‘Any beds, any beds?’ . . . he died on the Sunday morning, on his birthday. PC12 (wife)

Hospice staff also had to gauge how patient-carer needs changed over time, respecting that some couples wanted more marked boundaries, especially at the very end of life:

Well, I didn’t particularly want it. I know it sounds stupid, I didn’t want people coming in and out all the time, do you know what I mean? CC13 (wife)

Finally, a key gap in carer support was at the point of death, where carers experienced the ‘double grief’ of not only losing the person they care for but also the staff they had relied on:

The one thing I found hard is [husband] passed away, the girls [hospice carers] left and that was it. Now, you’re very busy at first. . . we had the funeral, we did all the form filling and then suddenly, I’m on my own. LC29 (wife)

Hospice bereavement teams did follow-up with a telephone call or letter (6 weeks–6 months post-death) but this rarely matched carers’ preferences and when the onus was on the carer to contact the hospice, most did not initiate. Some carers only wanted to speak to staff who they knew:

They’re aware of what you’ve been through so it’s easier to talk to somebody. . . that’s empathetic towards the situation that you’re in and that you’ve been through than it is to a complete stranger. VC09 (wife)

Post-bereavement services were varied but many carers did not engage in activities and/or did not find them suitable.

### Volunteer roles

All hospices relied heavily on volunteers to carry out a range of activities and acknowledged that volunteers were essential for running the hospice and longer-term sustainability (‘keeping it going’). Only one site used a small number of ‘care volunteers’ specifically trained to support hospice-at-home services to provide hands-on care, always with another employee but most staff expressed reservations about using volunteers at end-of-life. Concerns included reliability, risk management, governance and maintaining professional boundaries:

There’s a risk to our reputation. . . . . . the boundaries would need to be very, very clear before we introduced volunteers. VSP04 (Director of Operations)

However, the potential value of volunteers in hospice-at-home was acknowledged:

There’s an untapped resource we could use there and so many of our volunteers have the skills that could be developed into the clinical volunteer role. VSP03 (Chief Executive)

This might be pertinent to carers with limited social support who struggled with the burden of caring but were reticent to request support:

I did feel very much alone but then maybe it was my fault it. . . maybe if I had asked I might have got, I don’t know. PC05 (wife)

However, some carers also expressed reservations about involving volunteers:

I was getting very tired. . . they said that they could have someone to come and sit with [him] if I wanted to go somewhere. . . but I don’t know if he would have been comfortable, you know. . . a stranger coming in. GC18 (partner)

One service had surveyed carers and found that they wanted non-medical support that could be provided by volunteers and had instigated a pilot project to provide practical support, light housework and befriending. This appeared to boost carer confidence and reduce contact with paid staff, with volunteers acting as a bridge between home and hospice:

These volunteers were making a big difference. . . rather than the carer ringing up and talking to our palliative care nurses and taking a lot of medical time off, they would ring the volunteers. They felt more supported. DSP05 (volunteer co-ordinator)

## Discussion

### Main findings

Findings from the VOICES-Short Form were comparable with the most recent national data,^25^ which found that 79% of bereaved carers rated the overall quality of end-of-life care provided at home for their relative as outstanding, excellent or good. In our study, hospice-at-home services varied considerably, not just by our criteria (small/large, 24 h or not) but by level of hands-on care (reflecting differences in balance of qualified staff to healthcare-assistants), length of involvement (from hours to over a year) and variations in bereavement support. What stood out was the importance of staff having time to care (literally) and carers’ perceptions that they were unhurried; alongside this, hospice staff stood out for their expertise and working with the patient-carer dyad (‘Knowledge, skills and ethos’). Hospice-at-home staff were skilled in working with other services (e.g. district nursing) and managing territorial landscape (‘Integration and co-ordination’). Larger services tended to be involved for longer but provided less hands-on care. They were also able to provide earlier interventions but making early contact and placing the onus on carers to seek help when needed was not found to be supportive (‘Support directed at the carer’).

### What this study adds

We have provided novel insights into what carers perceive as ‘good’ care. It appears critical that hospice-at-home staff are not ‘generic’ workers but have the ‘knowledge, skills and ethos’ of highly trained specialist staff. Hughes et al.’s^26^ systematic review also highlighted the personal/professional qualities of hospice staff and their strong rapport with families. We found no evidence to suggest that general community nursing or care agencies had the time or expertise needed to match hospice staff. However, this highlights an inequity between community nursing, with limited capacity and expertise but who provide most community end-of-life care, and time-rich, highly skilled, hospice staff who only treat a fraction of those requiring input. This relates to the capacity of hospice-at-home services, under the programme theories of ‘marketing and referral’ and ‘sustainability’,^10^ reported elsewhere.^10^

Expertise was strongly associated with trust, in that carers expected any input under the umbrella of the hospice to be high quality (‘knowledge, skills and ethos’). Carvajal et al.’s^27^ review identified the importance of developing a relationship based on trust while Hughes et al.’s^26^ review identified the importance of a flexible and proactive service. We found if carers trusted hospice staff, who responded rapidly when required, carers were more likely to expect that the service could meet their needs (‘Support directed at the carer’). However, this was not always possible, highlighting the importance of setting clear expectations^27^ about what could (and could not) be provided and for how long, including abrupt withdrawal at the point of death.

Given limited resources, hospices need to consider how to develop their service model, one option being to include volunteers as part of the workforce working in the home, albeit with stringent processes to safeguard all parties. Volunteers could be used to provide support with domestic tasks (as in the COVID-19 pandemic), patient care when the volunteer has a relevant professional background, or more akin to the model of Compassionate Communities.^28^ This also has the potential for providing continuity of support post-bereavement.

### Strengths and limitations of the study

This study provides in-depth insights on hospice-at-home services across England, based on the views of carers and service providers. We acknowledge study limitations: data collection relied heavily on family carers and we were unable to recruit patients who did not have a carer; we were unable to provide translation services so could not recruit non-English speaking carers; we omitted to gather data on participants’ ethnicity, a significant oversight; and the number of patients recruited by some services was low, with one service dominating data for Large 24/7 services.

## Conclusion

Key markers of a good service included staff experienced in death/dying with time to care and provide hands-on care; who worked closely with other services to respond rapidly and provide the necessary intensity of care; whose knowledge and behaviour promoted supportive relationships through the process of dying and attended to carers’ needs. Larger services were able provide earlier interventions and a wider breadth of services. Areas of potential improvement included bereavement care and the use of volunteers in the home.

## Supplemental Material

sj-pdf-1-pmj-10.1177_02692163231206027 – Supplemental material for Family carer experiences of hospice care at home: Qualitative findings from a mixed methods realist evaluation Click here for additional data file. Supplemental material, sj-pdf-1-pmj-10.1177_02692163231206027 for Family carer experiences of hospice care at home: Qualitative findings from a mixed methods realist evaluation by Vanessa Abrahamson, Patricia Wilson, Stephen Barclay, Charlotte Brigden, Heather Gage, Kay Greene, Ferhana Hashem, Rasa Mikelyte, Melanie Rees-Roberts, Graham Silsbury, Mary Goodwin, Brooke Swash, Bee Wee, Peter Williams and Claire Butler in Palliative Medicine

sj-pdf-2-pmj-10.1177_02692163231206027 – Supplemental material for Family carer experiences of hospice care at home: Qualitative findings from a mixed methods realist evaluation Click here for additional data file. Supplemental material, sj-pdf-2-pmj-10.1177_02692163231206027 for Family carer experiences of hospice care at home: Qualitative findings from a mixed methods realist evaluation by Vanessa Abrahamson, Patricia Wilson, Stephen Barclay, Charlotte Brigden, Heather Gage, Kay Greene, Ferhana Hashem, Rasa Mikelyte, Melanie Rees-Roberts, Graham Silsbury, Mary Goodwin, Brooke Swash, Bee Wee, Peter Williams and Claire Butler in Palliative Medicine

sj-pdf-3-pmj-10.1177_02692163231206027 – Supplemental material for Family carer experiences of hospice care at home: Qualitative findings from a mixed methods realist evaluation Click here for additional data file. Supplemental material, sj-pdf-3-pmj-10.1177_02692163231206027 for Family carer experiences of hospice care at home: Qualitative findings from a mixed methods realist evaluation by Vanessa Abrahamson, Patricia Wilson, Stephen Barclay, Charlotte Brigden, Heather Gage, Kay Greene, Ferhana Hashem, Rasa Mikelyte, Melanie Rees-Roberts, Graham Silsbury, Mary Goodwin, Brooke Swash, Bee Wee, Peter Williams and Claire Butler in Palliative Medicine

sj-pdf-4-pmj-10.1177_02692163231206027 – Supplemental material for Family carer experiences of hospice care at home: Qualitative findings from a mixed methods realist evaluation Click here for additional data file. Supplemental material, sj-pdf-4-pmj-10.1177_02692163231206027 for Family carer experiences of hospice care at home: Qualitative findings from a mixed methods realist evaluation by Vanessa Abrahamson, Patricia Wilson, Stephen Barclay, Charlotte Brigden, Heather Gage, Kay Greene, Ferhana Hashem, Rasa Mikelyte, Melanie Rees-Roberts, Graham Silsbury, Mary Goodwin, Brooke Swash, Bee Wee, Peter Williams and Claire Butler in Palliative Medicine

sj-pdf-5-pmj-10.1177_02692163231206027 – Supplemental material for Family carer experiences of hospice care at home: Qualitative findings from a mixed methods realist evaluation Click here for additional data file. Supplemental material, sj-pdf-5-pmj-10.1177_02692163231206027 for Family carer experiences of hospice care at home: Qualitative findings from a mixed methods realist evaluation by Vanessa Abrahamson, Patricia Wilson, Stephen Barclay, Charlotte Brigden, Heather Gage, Kay Greene, Ferhana Hashem, Rasa Mikelyte, Melanie Rees-Roberts, Graham Silsbury, Mary Goodwin, Brooke Swash, Bee Wee, Peter Williams and Claire Butler in Palliative Medicine

## References

[1] NHS England. Ambitions for Palliative and End of Life Care: a national framework for local action 2021–2026. National Palliative and End of Life Care Partnership, https://www.england.nhs.uk/publication/ambitions-for-palliative-and-end-of-life-care-a-national-framework-for-local-action-2021-2026/ (2021, accessed 13 April 2023).

[2] Department of Health. End of Life Care Strategy: promoting high quality care for all adults at the end of life, https://www.gov.uk/government/publications/end-of-life-care-strategy-promoting-high-quality-care-for-adults-at-the-end-of-their-life (2008, accessed 21 April 2023).

[3] Hoare S Morris ZS Kelly MP , et al. Do patients want to die at home? A systematic review of the UK literature, focused on missing preferences for place of death. PLoS One; 10: e0142723. 10.1371/journal.pone.0142723 PMC4640665 26555077

[4] World Health Organisation (WHO). Integrating palliative care and symptom relief into primary health care: a WHO guide for planners, implementers and planners, https://www.who.int/publications/i/item/integrating-palliative-care-and-symptom-relief-into-primary-health-care (2018, accessed 19 July 2023).

[5] Office for Health Improvement and Disparities. Palliative and End of Life Care place of death factsheets, https://fingertips.phe.org.uk/profile/end-of-life (2023, accessed 14 April 2023).

[6] Keeble E Scobie S Hutchings R. The role of hospice services across the UK, https://www.nuffieldtrust.org.uk/research/support-at-the-end-of-life (2022, accessed 4 October 2022).

[7] Stokes L Holmes S. Hospice accounts. Analysis of the accounts of UK charitable hospices for the year ended 31 March 2021, https://www.hospiceuk.org/publications-and-resources/hospice-accounts-report-2022 (2022, accessed 13 April 2023).

[8] Woodman C Baillie J Sivell S. The preferences and perspectives of family caregivers towards place of care for their relatives at the end-of-life: a systematic review and thematic synthesis of the qualitative evidence. BMJ Support Palliat Care 2016; 6: 418–429. 10.1136/bmjspcare-2014-000794 PMC5256384 25991565

[9] Hashem F Brigden C Wilson P , et al. Understanding what works, why and in what circumstances in hospice at home services for end-of-life care: applying a realist logic of analysis to a systematically searched literature review. Palliat Med 2020; 34: 16–31. 31849270 10.1177/0269216319867424

[10] Butler C Wilson P Abrahamson V , et al. Optimum models of hospice at home services for end-of-life care in England: a realist-informed mixed-methods evaluation. Health Social Care Delivery Res 2022; 10: 1–304. 36063480

[11] Wong G Greenhalgh T Westhorp G , et al. RAMESES publication standards: realist syntheses. BMC Med 2013; 11: 21. 23360677 10.1186/1741-7015-11-21 PMC3558331

[12] Butler C Brigden C Gage H , et al. Optimum hospice at home services for end-of-life care: protocol of a mixed-methods study employing realist evaluation. BMJ Open; 8: e021192. 10.1136/bmjopen-2017-021192 PMC5961564 29769257

[13] Bell G Greene K Hunt J , et al. Developing national standards for hospice-at-home services. End Life J 2012; 3: 1–5.

[14] May CR Finch T Ballini L , et al. Evaluating complex interventions and health technologies using normalization process theory: development of a simplified approach and web-enabled toolkit. BMC Health Serv Res 2011; 11: 245. 21961827 10.1186/1472-6963-11-245 PMC3205031

[15] Rees-Roberts M Williams P Hashem F , et al. Hospice at home services in England: a national survey. BMJ Support Palliat Care 2021; 11: 454–460. 10.1136/bmjspcare-2019-001818 PMC8606452 31722982

[16] Yin RK. Case study research: design and methods. 5th ed. London: Sage, 2014.

[17] Addington-Hall J McCarthy M. Regional Study of Care for the Dying: methods and sample characteristics. Palliat Med 1995; 9: 27–35. 7719516 10.1177/026921639500900105

[18] Barker RJ Wilson P Butler C . How can the priorities of older, frail patients and their carers be used to inform policy and practice at the end of life? Insights from qualitative research across multiple settings. BMJ Open; 13: e068751. 10.1136/bmjopen-2022-068751 PMC10032383 36944473

[19] Pawson R. Digging for nuggets: how ‘bad’ research can yield ‘good’ evidence. Int J Social Res Methodol 2006; 9: 127–142.

[20] Jagosh J. Retroductive theorizing in Pawson and Tilley’s applied scientific realism. J Crit Realism 2020; 19: 121–130.

[21] De Weger E Van Vooren NJE Wong G , et al. What’s in a realist configuration? Deciding which causal configurations to use, how, and why. Int J Qual Methods 2020; 19: 1609406920938577.

[22] Gilmore B McAuliffe E Power J , et al. Data analysis and synthesis within a realist evaluation: toward more transparent methodological approaches. Int J Qual Methods 2019; 18: 1609406919859754.

[23] Wong G. Data gathering in realist reviews: looking for needles in haystacks. London: Sage, 2018.

[24] Allsop MJ Ziegler LE Mulvey MR , et al. Duration and determinants of hospice-based specialist palliative care: a national retrospective cohort study. Palliat Med 2018; 32: 1322–1333. 29874525 10.1177/0269216318781417

[25] Office for National Statistics. National Survey of Bereaved People (VOICES): London, England. 2015, pp. 1–20.

[26] Hughes NM Noyes J Eckley L , et al. What do patients and family-caregivers value from hospice care? A systematic mixed studies review. BMC Palliat Care 2019; 18: 18. 30736788 10.1186/s12904-019-0401-1 PMC6368799

[27] Carvajal A Haraldsdottir E Kroll T , et al. Barriers and facilitators perceived by registered nurses to providing person-centred care at the end of life: a scoping review. Int Pract Dev J 2019; 9: 1–22.

[28] Aoun SM Breen LJ White I , et al. What sources of bereavement support are perceived helpful by bereaved people and why? Empirical evidence for the compassionate communities approach. Palliat Med 2018; 32: 1378–1388. 29754514 10.1177/0269216318774995 PMC6088515

