# Supplementary material for: Family carer experiences of hospice care at home: Qualitative findings from a mixed methods realist evaluation
Source: Palliat Med. 2023 Oct 21;37(10):1529–39. doi: 10.1177/02692163231206027 (PMC10657508; doi:10.1177/02692163231206027)
Supplement: sj-pdf-1-pmj-10.1177_02692163231206027 – Supplemental material for Family carer experiences of hospice care at home: Qualitative findings from a mixed methods realist evaluation [file sj-pdf-1-pmj-10.1177_02692163231206027.pdf]

Supplementary file 1: Interview guide for staff

Introduction
How long have you worked for the hospice-at-home service?
How many years of service have you had as XX?
What did you do before working for the hospice-at-home service?

Let's talk first about how the service was developed:

(a) Do you know where did the idea of the hospice-at-home service come from?
(b) What makes it different to the current hospice service(s)?
(c) What makes your hospice-at-home service different to other End of Life Care (EOLC) services in the locality?
(d) How effective do you think local GPs are at talking to people about place of care and death?
(e) How much input did commissioners have into how the service was shaped?
(f) Do you think the marketing works?
(g) How do patients and families hear about the hospice-at-home service and about the care offered? Is there any information on the web?
(h) Can patients and families self-refer and how do you find that works?

What were the main aims or vision for the service when it started?

(a) What are the main benefits of the service?
(b) How does it add to existing provision?
  - are there any regional professional forums for the hospice-at-home services?
  - are there any networks (informal/formal) where you are able to share from practice through lessons learnt?

Let's now think how the service currently runs:

(a) How many staff are available (NB. use survey data to prompt)?
(b) What is the level / are the characteristics of service you provide i.e. 24/7?
(c) What do staff roles involve and what is the skills mix?
(d) What are the leadership and management arrangements for this service in your hospice? Does it have good senior support and championing? How proactive is the hospice-at-home manager?
(e) Who else do you work alongside? And how well does this work? How is the service integrated with other teams in the locality?
(f) Is there anything that "stands out" or is particularly innovative about your service that you are proud of?
    – if yes, please would you describe it?
(g) How do volunteers contribute to the hospice-at-home service?

Have there been any teething problems or issues with the service?

(a) How well does the hospice-at-home service work with other local services? What processes are in place to support better collaboration?

(b) How about linking up with other services – does your hospice-at-home work with other services *for example* in sharing patient records/EPaCCS (Electronic Palliative Care Co-ordination Systems/sharing a single point of access/sharing funding?<br>(c) Do you know if everyone is happy to work with one another across different services *for example* with handing over/sharing patient records?
How well is the service resourced?<br>(a) Do you think there are adequate staff numbers, adequate funding?<br>(b) What is the proportion/ratio of permanent staff, bank / agency staff or flexi staff? Will phasing out of zero hours contract impact on your hospice-at-home service?<br>(c) How does the hospice-at-home respond to unpredictable fluctuations in workload?<br>(d) Are there opportunities for further staff training?<br>(e) Are there problems with the recruitment and retention of staff – both front-line staff and service managers?<br>(f) What are the recruitment and retention strategies at your hospice-at-home?<br>(g) Are there issues around patients having to wait to access the service?
Let's now focus on the day to day work of the service:<br>(a) When patients are first referred into the hospice-at-home service, could you describe what happens?<br>- how appropriate are referrals as a whole?<br>(b) Would you be able to describe an 'average' day?<br>(c) Could you remember a particular case when you felt really good care was provided:<br>- what made this 'good' care?<br>- how about any care provided when you felt there could have been an improvement?<br>(d) Have you had any thoughts about how the Personal Health Budgets for EOLC might impact on the hospice-at-home service?<br>- do patients have a written care plan or how do you provide a guide for care they need?<br>(e) In your view, do you think the hospice-at-home is able to facilitate timely access to equipment and medications to patients?<br>(f) What is your experience and views on how the hospice-at-home service makes arrangements for anticipatory prescribing and care planning?<br>(g) How are carers supported by the hospice-at-home service?<br>(h) Do you have a positive example of working well with carers?<br>(i) Do you feel carers are overburdened? Why do you think that is?<br>(j) Are carers taught how to carry out medication administration?
How do you benchmark your service against others – both regionally and nationally?<br>(a) What information / measures are you collecting about hospice-at-home and what do you think of them:<br>- quality measures

- audit and evaluation<br>- 360 degree evaluation of the service<br>- satisfaction scores<br>- experience of service use<br>(b) Can you give me an example of how it's helped to improve practice?<br>(c) When you first spot a problem in the service, how is it tackled?<br>- would you be able to describe a case and how the problem was sorted out<br>- how did you find out that the problem had been resolved (i.e. monitoring)
How would you say the service has changed over the time you have known it?<br>(a) On reflection, how has the hospice-at-home service changed since it started?<br>(b) How is the hospice-at-home model organised? Are there hubs or a single point of access?<br>(c) How adept has the service been in responding to changes in things like local need (i.e. demographic, clinical etc.), funding type or becoming more integrated with other services
