# Supplementary material for: Family carer experiences of hospice care at home: Qualitative findings from a mixed methods realist evaluation
Source: Palliat Med. 2023 Oct 21;37(10):1529–39. doi: 10.1177/02692163231206027 (PMC10657508; doi:10.1177/02692163231206027)
Supplement: sj-pdf-2-pmj-10.1177_02692163231206027 – Supplemental material for Family carer experiences of hospice care at home: Qualitative findings from a mixed methods realist evaluation [file sj-pdf-2-pmj-10.1177_02692163231206027.pdf]

Supplementary file 2: Additional post-bereavement questions

Q1. Overall, do you feel that you and your family got as much help and support from health and social care services as you needed when caring for him/her? (tick appropriate answer)

- ☐ Yes, we got as much support as we wanted
- ☐ Yes, we got some support but not as much as we wanted
- ☐ No, although we tried to get more
- ☐ No, but we did not ask for more help
- ☐ We did not get any help at all
- ☐ Not applicable, we did not need any help

Q2. Overall, do you feel that the help and support you and your family received from health and social care services when caring for him/her was: (tick appropriate answer)

- ☐ Outstanding
- ☐ Excellent
- ☐ Good
- ☐ Fairly good
- ☐ Poor
- ☐ Not applicable, we didn't need any help
