# Supplementary material for: Family carer experiences of hospice care at home: Qualitative findings from a mixed methods realist evaluation
Source: Palliat Med. 2023 Oct 21;37(10):1529–39. doi: 10.1177/02692163231206027 (PMC10657508; doi:10.1177/02692163231206027)
Supplement: sj-pdf-3-pmj-10.1177_02692163231206027 – Supplemental material for Family carer experiences of hospice care at home: Qualitative findings from a mixed methods realist evaluation [file sj-pdf-3-pmj-10.1177_02692163231206027.pdf]

Supplementary file 3: Carer topic guide

Let's first begin with when X (relative / spouse/ parent / other) started to use the hospice-at-home service:

(a) What problems were you and X experiencing before the hospice-at-home started?
(b) After the hospice-at-home service started, were you aware which staff and what care was being provided by the hospice-at-home service, and by any of the other services?
(c) What impact did the hospice-at-home service have on X and you in your situation?

Turning to think about the key contacts / most helpful people:

(a) Who do you think were the main people who saw X and you when receiving care from hospice-at-home?

Let's focus on how you found the hospice-at-home service:

(a) What parts of the service helped?
   - in what ways
(b) Did you feel the care X and you received was joined up in any way?
   - if yes, in what ways?
   - if no, why do you think this was the case?
(c) Were there elements of the service that helped X and you particularly? For example:
   - working together when handing over to a new member of staff
   - always having one person at hand to talk
(d) What parts of the service were less helpful?
   - in what ways

Let's think about the types of care X received:

(a) Can you remember what care X received?
   - how did this help?
   - was there anything else you thought was needed?
(b) How did the hospice-at-home service compare with other services you received?
   - what was different?
   - what was better?
   - what required improvement?

How well did hospice-at-home take into account X and your wishes:

(a) How confident / safe did you feel with the service?
(b) Did you feel the service understood what you both wanted?
   - did you feel included in the care
   - did you feel supported emotionally
   - did X die in the circumstances they wanted to? If not, why do you think this is?

Let's focus how hospice-at-home worked for you and X on a day-to-day level:

(a) Did you feel the staff were able to do what was required?
  - i.e. the 'right' care at the 'right' time
  - examples of team working
  - examples of care being provided with the staff working together

(b) Were you able to contact the hospice-at-home service when you needed them?
  - enough time given to you
  - more or less contact time required?

Let's think about the help *you* received from hospice-at-home:

(a) Would you be able to describe in your own words the support you received from the hospice-at-home service when caring for X?

(b) Did the service continue to meet your expectations?

(c) Were there any other issues that came up *for example* gaps in care, things you needed but could not get?

(d) Did you feel the service relied on you a lot?
  - were you happy with this?

(e) Do you think you could have managed at home without the service?

Turning to think about how hospice-at-home takes on board feedback:

(a) Has anyone approached you to feedback about the service?

(b) What would you say was positive about using the service?

(c) Can you suggest any ways in which the service could do anything better?

(d) Did you find the service adjusted to your needs as you went along?
  - i.e. did you feel listened to?

To end with, let's focus about your overall experience of the hospice-at-home service:

(a) What do you feel the service offered you and X?

(b) What was distinctive if anything about the hospice-at-home service compared to other services?

(c) Was this something more in terms of quantity or quality than you were receiving from other services?

(d) In such difficult times, what were you hoping hospice-at-home would provide? And were your hopes achieved?
