# Supplementary material for: Family carer experiences of hospice care at home: Qualitative findings from a mixed methods realist evaluation
Source: Palliat Med. 2023 Oct 21;37(10):1529–39. doi: 10.1177/02692163231206027 (PMC10657508; doi:10.1177/02692163231206027)
Supplement: sj-pdf-4-pmj-10.1177_02692163231206027 – Supplemental material for Family carer experiences of hospice care at home: Qualitative findings from a mixed methods realist evaluation [file sj-pdf-4-pmj-10.1177_02692163231206027.pdf]

Supplementary Information: Full Context–Mechanism–Outcome Configuration Tables, Version 25

(Butler et al., 2022, Appendix 30, Table 92)

N.B. Columns with transcript and page numbers have been removed.

1 – SUSTAINABILITY

**CONTEXT**<br>**The backdrop, this may vary in different case study sites allowing mechanisms to trigger or not**	**MECHANISM**<br>**The causal action(s) and responses to those actions which achieve the outcome in the context**	**OUTCOME**<br>**These will be desirable but there may also be undesirable outcomes (red font) produced by the same contexts and mechanisms.**
**FUNDING**<br>**Statutory funding conditions and arrangements change over time i.e. the constantly changing landscape of commissioning structures and provider organisations' arrangements.**	**The H@H service needs to "be on the front foot", i.e. if the H@H service proactively seeks visibility, a seat at the table and control over the available statutory funding by one or more approaches.**<br><br>**AND/OR**<br><br>**The H@H organisation actively seeks external engagement with the wider health and social care environment.**<br><br>**MECHANISM RESPONSE**<br><br>**The H@H service is trusted and respected (based on its reputation) to know what**	**Sustainable, longer-term funding is enabled and patients will continue to receive the H@H service.**

	**services are needed, to raise funds to deliver them and then to deliver them.**<br><br>**AND/OR**<br><br>**If the H@H service is run by an independent organisation which undertakes charitable fundraising from different sources**	
**<u>COMMISSIONING</u>**<br>**<u>There is often difficulty establishing consistent relationships with commissioners</u>.**<br><br>**<u>Commissioners may not be knowledgeable about palliative and end of life care</u>.**<br><br>**(Commissioners change frequently and very few are in post long enough to develop a good understanding of palliative and end of life care services, including National initiatives.)**<br><br>**<u>Commissioners may not recognise the full "cost" of what they are commissioning</u> as significant charitable funds are supporting the H@H service.**	**Commissioners new to EOLC with little history of a relationship with the H@H may not be aware of the service or have had time to develop trust in the service**	**Access to statutory funds may be compromised.**

**STAFFING**<br>**There is a shortage of staff in health and social care nationally.**<br><br>**Some H@H services do not pay salaries compatible with statutory NHS/social care rates.**<br><br>**Some affluent areas may be too expensive for lower paid staff to afford to live there.**<br><br>**Some rural areas may have even more limited workforce availability.**	**The reputation of the H@H organisation for excellence and investment in staff through CPD, and the hospice/charity ethos attract staff, and even outweigh considerations of lower pay.**<br><br>**Skill mix profiles may be altered in response to RN shortages and registered staff deployed differently (e.g. office-based, dealing with triaging and work allocation)**	**The H@H service is able to recruit the staff they need to deliver the care that patients need**<br><br>**The H@H service may attract and retain staff from other services depleting the workforce in other parts of the system.**<br><br>**Staff take on roles they are not able to manage (in terms of skills and training).**<br><br>**Staff may not be doing their preferred work.**
**NATIONAL POLICY**<br>**Commissioning has responsibility for whole population needs and care.**<br><br>**There is a national drive towards care at home, ostensibly in response to patient “choice”, but also with a view to cost savings and reducing pressure on hospital beds.**	**Home based care is supported by local health and social care commissioning and funding.**	**Rolls Royce service for some, none for others**<br><br>**Policy supports the provision of home-based care which suits those who want to die at home.**

There are also societal and family pressures suggesting that "home is best".<br><br>AND<br><br>Hospice inpatient beds are a relatively scarce commodity (Goldilocks quote).	If hospice at home services are available in the area and offered to patients and families and their wishes and preferences are not fully explored and revisited over time.	Patients may feel they have limited options and dying at home is what is expected.
<u>DATA AND "EVIDENCE"</u><br>NHS commissioners and charity boards require the collection of data to provide "evidence" to support continuing service provision and development.	Enormous volumes of activity data are collected. Very little outcome data or cost benefit data is collected or derived (e.g. about avoided hospital admissions).	Activity data may satisfy some stakeholders.<br><br>Time is wasted in data collection which cannot richly inform funding and service decisions.<br><br>May lead to staff frustration and resistance.

2: Volunteers

CONTEXT	MECHANISM<br>The causal action(s) and responses to those actions which achieve the outcome in the context	OUTCOME
**There are national workforce shortages in health and social care so that the paid workforce is in short supply.**<br>**AND**<br>**Societal norms re neighbours, community behaviour, families living in close proximity etc. have changed.**<br>**AND**<br>**Many people in communities offer their time and skills as volunteers to hospices and other organisations and** recruiting, training and managing volunteers takes considerable time<br>**AND**<br>**Some of these people may have relevant health and social care professional skills.**<br>**THEREFORE**<br>**The volunteer workforce is attractive to employers but hospices may be concerned about utilising a volunteer workforce, particularly in the clinical setting, feeling that it is not as manageable or reliable as the paid workforce, and having concerns about legal liability, health and safety etc.**	**If H@H organisations invest in people and systems to recruit and manage volunteers thereby reassuring the hospice organisation about working with a volunteer workforce.**<br><br>**If volunteers with existing, relevant skills are identified and they are prepared to use them in the H@H service.**<br><br>**AND**<br><br>**If volunteers have** roles, remit, boundaries and expectations which are **clear.**<br><br>**If volunteers are well supported by the organisation in their role.**<br><br>**OR**<br><br>**If the organisation or locality takes a different approach to volunteering (ref compassionate communities), tolerating a different level of "risk" and allowing volunteers to act more like neighbours**	**Then the hospice will feel confident to utilise a volunteer workforce and additional resources will be available to provide care and support to patients and families**<br><br>**Volunteers feel confident and clear in their role, volunteer well-being is facilitated and volunteers are retained within the H@H to provide enhanced care for patients and families.**<br><br>**Then volunteers will be able to maintain appropriate boundaries that safeguard the patient, their family and the volunteer.**<br><br>**More volunteers may be able to get involved with caring and supporting people at home.**<br><br>**HOWEVER**

	without a great deal of bureaucracy and procedure.	Volunteers may find the structure and expectations too demanding and inflexible and chose to volunteer elsewhere.<br><br>Inconsistencies, paradoxes and tensions develop when setting multiple "rules" for volunteers who are offering non-professional support and befriending. They cannot in reality hold both a "semi-professional" and a befriending role.
Where H@H services offer shorter periods of care, in situations where the physical care needs are significant and rapidly increasing.	Volunteers cannot normally be trained and managed to meet the predominant healthcare needs when the patient is actively dying.<br>There may be exceptions here in terms of volunteers with particular skills (e.g. retired nurses).<br><br>Volunteers could be utilised specifically to support the carer thus enabling the whole home situation to be sustained (e.g. doing the laundry, collecting prescriptions).	Volunteers in general may have a limited role in this element of hospice volunteering.
Hospices rely heavily on the volunteer workforce to support fundraising activities including charity shops, sponsored events etc.	The arrangements for volunteers may be more straightforward and easier to manage in this context.	Volunteers make a valuable contribution to fundraising, without which services would be more limited.

3: Raising awareness, service profile, criteria and referral

CONTEXT	MECHANISM<br>The causal action(s) and responses to those actions which achieve the outcome in the context	OUTCOME
**RAISING AWARENESS**<br>There is a complex system of health and social care providing services for patients in the community at end of life and many professionals are not aware of all the possible services or of details of patient suitability.<br><br>In particular, hospice services are often thought of as hospice buildings and there is less understanding of hospice community services.	The H@H actively raises awareness to professionals and the public through clinical and public engagement:<br><br>- Raising awareness to the public (via phone number, website, GP, previous experience of hospice services) and enabling them to "get a foot in the door"<br><br>- the fundraising element of the H@H organisation uses fundraising events also to market the service and educate the public about its role.<br><br>- Clinical staff have an important role in encouraging referral to the service through their day to day work and interactions with colleagues and the public	The H@H will be seen as an essential service by professionals and the public and more patients with suitable needs receive timely referral and care.<br><br>Raising awareness directly to the public overcomes some of the professional barriers to referral and improves access.

**<u>GETTING TO THE RIGHT PATIENTS AND EQUITY</u>**<br>**Not all patients who could benefit from H@H services are currently referred.**<br><br>**H@H services aim to offer care to patients with "complex" and suitable needs.**<br><br>**H@H services prefer to avoid discharging patients from services because of the impact on reputation and the difficulty of replacing H@H care; "we'd never leave someone in the lurch"**	**The H@H service proactively seeks suitable referrals through a range of systems or relationships, e.g. continuing healthcare pathways, hospital palliative care team relationships.**<br><br>**If the H@H has robust criteria for identifying which referrals are suitable, which may include trained triage staff to manage service admission.**<br><br>**Clearly boundaried funding arrangements (e.g. CHC funding) enable more robust management of accepting or discharging patients from the H@H service.**<br><br>**If criteria, explanation of the service and treatment are clearly communicated to patients, families and health and social care professionals.**<br><br>**If there is clear information about referral onto other services when the H@H service is not deemed suitable.**<br>**OR**	**Suitable patient referrals who can benefit most will receive the service.**<br><br>**Professionals, patients and families will be clear about when and what they can expect to receive from the H@H service leading to better managed expectations of the H@H service.**

	**Patients and carers accept reduced care at times when H@H resources are stretched. This is based on the quality of care received (they believe other agencies will not match it) and on the "charitable" ethos which pervades the services.**	
**Professionals may be reticent to refer patients to hospice at home services because it means having a conversation about death and dying with the patient which they may not feel skilled or willing or have enough time to do.**<br><br>**Referrers may think the service is only for cancer patients.**<br><br>**The public have negative perceptions of the hospice involving the fear and stigma of death and dying (may have more impact in some communities in particular).**	**If the H@H focuses its message of living well at all stages, this may make the process of referral more palatable for professionals and patients alike.**<br><br>**H@H services offer education and skills training about recognition of end of life, having end of life conversations and the use of the palliative care register.**	**More patients with suitable needs receive timely hospice at home care, including non-cancer patients (e.g. with dementia, frailty).**
**A number of patient groups are poorly represented in hospice services in general, i.e. do not receive an equitable share of available services, e.g. older age >85, BAME, non-cancer diagnosis, deprivation**	**The H@H raises awareness about its service to professionals and the public through clinical and public engagement (see detail above)**<br><br>**AND/OR**<br><br>**If the service includes a pro-active element to draw in suitable patients (e.g. Could**	**Caseload reflects suitable diagnostic, demographic, cultural & socioeconomic diversity of the area served and services are provided equitably.**

	**specifically target those groups locally known not to access the service by tailoring or directing marketing to those under-represented groups)**	
**Referrals to the H@H service require transfer of information which may be time consuming and require duplication of records and also effort finding the correct format and processes**	**If referral to the H@H is easy for clinicians to do; long and formal referral processes are a deterrent.**	**More patients with suitable needs receive timely hospice at home care**
**MANAGING EXPECTATIONS**<br>**The term "hospice" encourages expectations of quality and specialism which H@H services may not be able to replicate at home.**<br>**There are limits, including workforce shortages, to what can be provided in the home setting which cannot mirror exactly the provisions of an inpatient setting.**	**Patient and carer preferences are explored**<br>**AND**<br>**Criteria, explanation of the service and treatment are clearly communicated to patients, families and health and social care professionals.**	**Professionals, patients and families will be clear about when and what they can expect to receive from the H@H service.**<br>**AND**<br>**Patients make choices based on correct information and understanding and their choices are not overridden by professionals.**

4: Integration and co-ordination

CONTEXT	MECHANISM<br>The causal action(s) and responses to those actions which achieve the outcome in the context	OUTCOME
SERVICE PERSPECTIVE<br>A range of services needed by people at end of life are operating in the community with different organisational, funding, staffing, IT etc arrangements<br>AND<br>Services across the whole system commonly act in silos, resulting in both duplication and gaps in services received by patients.<br>AND<br>There is a limit to services, funding and workforce across the whole system.<br>AND<br>Issues of professional "ownership" of palliative and end of life care are at play whereby designated palliative care services may both wish to see other services providing end of life care but also see this as a threat.<br>AND<br>Professional boundaries are shifting across health and social care including those between doctors, nurses and other professionals (paramedics, physician	The H@H service is working effectively with other service providers internally, externally and on the ground.<br>Coordination between workers on the ground is pivotal.<br>COMMUNICATION is a key mechanism here:<br><br>e.g. a blended service is provided whereby different services can provide what is needed by the patient without hard boundaries around particular roles; honorary contracts with NHS are emblematic and may facilitate this.<br><br>e.g. A secondment into a different setting (e.g. a healthcare worker into social care) may facilitate integration by the "learning of another language" (dependent on workforce availability)	Patients and carers receive a seamless service & continuity of care without delay, duplication or gaps, e.g. care, interventions, equipment and drugs that are needed by the patient will be available in a timely fashion.<br><br>More cost effective services are delivered to patients and families.<br><br>Less burden on H@H staff trying to ensure coordination and reduced tension between care providers<br><br>BUT<br><br>The multiplicity of organisations and roles may cause confusion and conflict ("ruffled feathers").

<table>
<tr>
<td rowspan="2">associates) and between registered and non-registered workers.
AND
Integration of health and social care is a national driver, the boundaries between the two in terms of care needs are often difficult to define clearly and many years have been spent creating/putting in divides between them for the purposes of previous funding divisions.
AND
Organisations seek their own branding and distinctiveness for sustainability purposes.
AND
Individual professionals seek to differentiate their roles and functions so they all continue to be "needed", for their own personal sustainability.
AND
Services will be covering a variety of urban and rural areas (majority mixed) and travelling time will be a significant factor in service delivery responsiveness and resources.</td>
<td>e.g. shared clinical records/IT systems (some examples of this but many areas are far from this)</td>
<td></td>
</tr>
<tr>
<td>EXAMPLES: MEDICATION AND EQUIPMENT
e.g. DNs provide and administer all anticipatory medications (agreed division of labour),

e.g. the H@H service may have medical or non-medical prescribers available in the H@H service,

e.g. the H@H service is trusted to make assessments which other professionals will act upon. N.B. this trust is based on individuals and/or on the reputation of the H@H service as a whole.

e.g. The H@H service has direct access to shared equipment stores or have their own stores</td>
<td>This specific example may result in unnecessary duplication when a qualified member of staff who is in the home cannot perform a task because it has been allocated to another service and requires a separate visit.</td>
</tr>
<tr>
<td>PATIENT AND FAMILY, NEEDS "AT THE FRONT DOOR"
Patients in the last phase of life and their family carers require and use services from</td>
<td>Regardless of "formal" arrangements for integration and coordination, much of this</td>
<td>Patients receive a seamless service & continuity of care with consistent</td>
</tr>
</table>

**a wide range of statutory, voluntary, health and social care providers.**<br>**AND**<br>**Palliative and end of life care patients have a constantly changing trajectory of illness and a high risk of unexpected and unpredictable needs which are difficult to anticipate.**<br><br>**Some patients and carers will not know when to ask for help or who to contact. Patients are not always clear about when and what they need that should trigger them making contact to request services. They prefer not to feel that they need hospice care (implies their situation is extremely serious). They don't want to make too many demands on busy and charitably funded services.**	**works on the ground as colleagues get on to work for the patient and are co-dependent.**<br><br>**If patients and carers are provided and updated with information including who and how to contact professionals (within H@H and with other organisations in the locality).**<br><br>**If budgets and workforce and organisational structure are managed in an integrated way across provider organisations.**<br><br>**e.g. an element of flexible workforce is employed (by the H@H service or others) or staff are flexibly deployed from other areas (e.g. IPU)**<br><br>**e.g. advance plans are made and the need for medications and equipment are anticipated provided on time.**	**information, without delay, duplication or gaps (they really don't care who is providing it!)**<br><br>**Services are able to provide staff to respond rapidly to unpredictable and fluctuating workloads in a cost effective way.**<br><br>**Patients know who to contact in an emergency and get the response they need**<br><br>**Fewer patients have unwanted emergency admissions**<br><br>**More patients have enhanced hospital discharge**
**IN HOURS vs OUT of HOURS SERVICES**<br><br>**Not all H@H services themselves provide 24/7 care.**	**Anticipatory care and information become yet more critical and in particular, if night time cover is not provided by H@H, a well-advertised 24h contact number is crucial.**	**Needs are anticipated as far as possible and patients experience continuity of care when providers change.**

5: Knowledge, skills and ethos of care providers

CONTEXT	MECHANISM<br>The causal action(s) and responses to those actions which achieve the outcome in the context	OUTCOME
<u>All health and social care workers should have basic knowledge and skills in palliative and end of life care.</u> However, these are sometimes lacking, e.g. lack of confidence in identifying the end of life, lack of skills in communicating at end of life and lack of time to offer personalised and patient-led care.<br><br><u>TIME</u><br>The provision of time is a context which H@H services aim to offer, to add value to the whole system of care.<br>They can pick and choose their workload more than services which are statutory or which are profit making.<br><br>Commissioners do not always value Time and Expertise but prioritise equity of access across the population.	H@H services provide expert knowledge and skills in palliative and end of life care and have a suitable ethos to support this care by:<br><br><u>EXPERIENCE</u><br>Experienced staff, spending a significant proportion of their time in palliative and end of life care, who are capable and competent in this setting (MECHANISM RESPONSE) so patients, families and other HCPs trust them.<br><br><u>TIME</u><br>Taking time to offer personalised, patient-led care, not having to rush (pacing).<br>Time pressures are well managed by H@H organisations through sensitive communication with patients and carers (e.g. if they are going to be delayed).	Better patient and carer experience and sense of agency.<br><br>More patients receive the care they need when they need it and where they prefer to be

There is wide variability within localities in the levels of skills, knowledge and performance of community based staff, which may vary GP to GP, community nurse to community nurse, practice to practice.<br><br>Palliative and end of life care has developed into a "specialty" area of knowledge, skills and ethos and this distinctiveness is prized by H@H organisations.<br><br>There is a range of other "specialists" also working in the community which will vary from one locality to the next. This may include: other palliative care staff, Admiral nurses, respiratory teams, heart failure services. They will also have varying levels of skills, knowledge and confidence in dealing with palliative and end of life care.<br><br>For some patients, basic skills may not be adequate to meet their difficult or complex needs.	TRAINING<br>staff at all levels (including volunteers) are suitably trained including appropriate communication skills and investment in CPD, so that they can create an environment (MECHANISM RESPONSE) making patients and carers feel they are in expert hands<br>The H@H service also provides training to other H&SC professionals.<br><br>SUPPORTED STAFF<br>The H@H retains skilled staff by providing staff support to manage the stress of their work and to develop the necessary emotional resilience.<br><br>Staff can call for advice and support with their work from expert colleagues who are part of the H@H service.<br><br>AND<br><br>policies and processes allow for extended roles as necessary (examples) and staff	Other health and social care workers may be upskilled in some instances or de-skilled in others. If the balance falls in terms of de-skilling, then knowledge and confidence decreases over time resulting in poorer care for those who do not access specialist services and increasing demand for specialist services.

	know who to collaborate with if work is outside of their competencies	
**Most of the expertise in palliative and end of life care still resides in cancer care and patients with other illnesses (e.g. dementia) may present particular challenges to staff and organisations**	**Accessing other elements of hospice services because prognosis is longer than H@H can provide for may support some of these patients.**	**Inequity and mis-match in care provided, patient/carer feel patronised and not understood.**

6: support directed at the carer <u>or</u> support directed at the whole patient/carer(s) dyad at home

CONTEXT	MECHANISM<br>The causal action(s) and responses to those actions which achieve the outcome in the context	OUTCOME
There is a societal expectation that "family" will support dying at home and services are configured on this basis. Unpaid care provided by family and/or friends is critical to enabling patients to remain at home.<br><br>Informal carers and the home circumstances vary in the extent to which they can or wish to support care at home. This will include cultural differences and preferences. The home "unit" needs to make a decision to aim to support death at home.<br><br>There will be a spectrum of needs and wishes of patients and carers and how they align with each other.<br><br>The carer requires continuing confidence, wellness (physical and emotional), and new skills to enable them to provide care up to and including the point of death at home. The task of caring at home is constantly changing over time.	If there is an assessment and continuous review of carer needs including the whole family and care unit and If there is a multidisciplinary team available to meet the needs.<br><br>How the patient and their informal carers as a unit in the home feel about caring at home and respond to the challenge of this situation will be key to sustaining the care.<br><br>If carer is informed as early as appropriate about what H@H can/cannot offer and how it fits in with other services<br><br>If carer is informed about and signposted to other services and community support (including services specifically directed at carers)<br><br>If H@H are able to 'coordinate' with other services and advocate for the patient if the carer unable	<u>Carers will be able to continue to care enabling more sustainable patient care at home.</u><br><br>Carers will receive the care and support they need, including sleep, taking a break, reassurance, confidence building<br>Carer is not isolated.<br><br>Occasionally, carer needs may become the main focus at the expense of the patient

**People have different views on their home and their willingness to admit outsiders; these views may differ between patient and carer.**	**If carer is informed about funding options and available financial support is accessed (eg carers allowance)**<br><br>**If cultural preferences are respected**<br><br>**If the carer is fully informed including what might happen in terms of the trajectory of illness and the increasing burden of caring over time, they will know what to expect and prepare and they can rapidly recognise a change in caring situation from control to crisis**<br><br>**If carer and patient choices are encouraged, affirmed and supported wherever possible,**<br><br>**If carer is offered respite services**<br><br>**If self-referral / referral by informal carer to H@H is made available and easy to follow**<br><br>**<u>Carer sets the pace.</u> If there is a negotiated partnership between the carer and staff (or NOT, examples) and recognition of what the individual**	**If too much is expected of the carer (e.g. administering medication injections when they prefer not to) there are some tasks they may not be able to manage or they may not be able to continue to provide care or they may have a negative or distressing experience.**<br><br>**The carer and patient feel supported and encouraged.**<br><br>**There is respect for the relationship between patient and carer who get the chance to spend quality time together.**

	**pt/carer dyad want (i.e. some may not want a lot of intrusion into their home)**<br><br>**AND**<br><br>**This is followed by an ongoing relationship of feedback, response and adaptation**<br><br><br>**If the carer has (quick) access to an appropriate point of contact 24/7**<br><br>**If there is regular contact with the carer**<br><br>**If carer is offered training/information on practical aspects of care (same moving and handling, medication administration, mouth care, preventing pressure sores)**<br><br>**If the procedures after death meet cultural needs and preferences (eg burial within 24 hours)**<br><br>**MECHANISM RESPONSE will be trust and confidence in the backup provided by the service and in their caring role.**<br><br><br>**Negotiations take place with the carer about how much they are happy to take on and the carer**	

	receives skills training so they will have appropriate skills that they find are acceptable.<br><br>Carer is recognised for their knowledge and given permission to do caring tasks which are increasingly seen as "professional" tasks<br><br>Carer and patient are supported to make advance care plans and post-death preferences	
**AFTER DEATH**<br>There may be short or long term consequences of caring to the carer's mental and physical health, in bereavement.<br><br>H@H services usually come to an abrupt halt when the patient dies.<br><br>The existing social networks that carers have vary considerably.	There is support pre- and post-bereavement which is based on relationship and understanding of the situation and also a shared story of caring for the patient.<br><br>There is flexible and varied post-bereavement support (e.g. support groups and one-to-one support) at the hospice and wider community and a way to keep in touch with the hospice (eg events)<br><br>There is carer-centred guidance available on practical tasks after death (e.g. death certification, funerals, legal advice and dealing with leftover equipment/medication/records)	The carer will have the best chance of a positive outcome following bereavement and recovery from the caring role.<br><br>The carer feels they have done their best for their loved one.<br><br>Carers may have a negative experience of care and the burdens and difficulties.<br>AND<br>Some carers may accept this negative experience as a price they wish to pay to allow their loved one to achieve their preferred place of death.

**There is a concern about "medicalising" bereavement which is a normal process.**	**There is a mechanism to identify those who are not experiencing "normal" bereavement and may need additional/expert help. Routine bereavement letters, memorial events may provide opportunities for this.**	
