# Supplementary material for: Family carer experiences of hospice care at home: Qualitative findings from a mixed methods realist evaluation
Source: Palliat Med. 2023 Oct 21;37(10):1529–39. doi: 10.1177/02692163231206027 (PMC10657508; doi:10.1177/02692163231206027)
Supplement: sj-pdf-5-pmj-10.1177_02692163231206027 – Supplemental material for Family carer experiences of hospice care at home: Qualitative findings from a mixed methods realist evaluation [file sj-pdf-5-pmj-10.1177_02692163231206027.pdf]

Supplementary file 5: Final programme theories

Integration and coordination
Services across the whole system commonly act in silos, resulting in both duplication and gaps in services received by patients. This is compounded by a limit to services, funding, and workforce. In addition, issues of professional ownership of end-of-life care are at play, and organisations seek their own branding and distinctiveness for sustainability purposes. Patients in the last phase of life often have unpredictable needs at times which are difficult to anticipate. Some patients and carers will not know when to ask for help or who to contact. The hospice-at-home service needs to work in a coordinated and effective way with other service providers. This may be through a blended service without hard boundaries around roles or services, a secondment into a different setting which facilitates integration, or an agreed division of labour between services. If patients and carers are provided and updated with information, including who and how to contact professionals, then the chances of them receiving a seamless service and continuity of care with consistent information increase.
Knowledge, skills and ethos of care providers
Whilst all health and social care workers should have basic knowledge and skills in end-of-life care, sometimes these are lacking, including a lack of confidence in communicating at end of life. For some patients, basic skills may not be adequate to meet their difficult or complex needs. Palliative and end of life care has developed into a specialty area of knowledge, skills and ethos, and this distinctiveness is prized by hospice-at-home organisations. However much of this expertise still resides in cancer care and patients with other illnesses, such as dementia, may present challenges to staff and organisations. Some services (hospice-at-home and non- hospice-at-home) may also lack time to offer personalised and patient led care, while commissioners may prioritise equity of access across the population rather than time and expertise. To add value to the whole system of care, hospice-at-home services need to provide expert knowledge and skills in end-of-life care with a suitable ethos to support this care. This is enabled by experienced staff who have spent a significant proportion of their time in EOLC so that patients and families trust them. Staff at all levels, including volunteers, are suitably trained including appropriate communication skills so that they can create an environment where patients and carers have confidence and feel they are in expert hands. Hospice-at-home services value the time to offer personalised patient led care, leading to better patient and carer experience and sense of agency.
Support directed at the carer or patient-carer dyad at home
Unpaid care provided by family and friends is critical to enable patients to remain at home. How the patient and their informal carer, as a unit in the home, feel about dying at home and respond to the challenge of this situation will be key to achieving death at home. The carer may require confidence and new skills to enable them to provide care up to and including the point of death at home. In bereavement, there may be short or long-term consequences of caring to the carer's mental and physical health. However, there is a concern about medicalising bereavement which is a normal process.<br>A full assessment of care needs including the whole family/care unit is required. The hospice-at-home service fully informs the carer about what might happen in terms of the trajectory of illness and the increasing burden of caring over time. Carers will then know what to expect and can rapidly recognise a change in caring situation from control to crisis. If carer and patient choices are affirmed and supported wherever possible, the carer and patient have an increased sense of control. The hospice-at-home service should negotiate a partnership with the carer, including clarity about what can and cannot be provided, and recognition of what the patient-carer dyad wants. Pre-and post-bereavement support is based on relationship and understanding of the situation, and a shared story of caring for the patient. In addition, those not experiencing normal bereavement need to be recognised and additional help made available. This should not rely on self-referral and the timing may be many months post bereavement.

Volunteers
Workforce shortages and the willingness of many people in the local community to volunteer makes the volunteer workforce attractive to hospice and palliative care organisations. Changing societal norms around family and community structure have impacted on the social networks of many patients and carers, with demand on carers compounded by hospice-at-home's limitations in providing longer periods of input. Whilst a volunteer workforce could result in extending this period of care, hospice-at-home need to effectively recruit, train, and manage volunteers including providing clear responsibilities, support and lines of reporting. However, to reduce the bureaucratic burden, the hospice-at-home may take a different approach to some aspects of volunteering, along the lines of the Compassionate Communities model with volunteers acting as good neighbours.
Marketing and referral
There is a complex system of health and social care providing end of life care for patients in the community. Furthermore, hospice services are often thought of as a building and there is less understanding of hospice-at-home services. Hospice-at-home functions in a society where there is a fear and stigma around death and dying, (particularly in some communities), with potential referrers reticent to have conversations with the patient regarding prognosis. To increase referrals in general, and in particular of those that are poorly represented in hospice services, hospice-at-home needs to actively market its service to professionals and the public through clinical and public engagement. Referral systems need to be as simple as possible and not require complex transfer of information.
Sustainability
Hospice-at-home services exist in an environment where there are constantly changing funding arrangements and commissioners, and an increasing requirement for data to provide evidence to support commissioning. There are also local and national shortages of health and social care staff, alongside a national drive towards care at home. For sustainable, longer-term funding within this context, the hospice-at-home needs to proactively seek control over available statutory funding, engage with the wider health and social care environment, and if a charitable organisation, to undertake fundraising and income generation from a range of sources. To recruit and retain staff to deliver the care that patients need, the hospice-at-home requires a reputation for excellence and investing in staff development and will alter skill mix profiles in response to local workforce shortages.
